# Association Between Skin Autofluorescence and Coronary Heart Disease in Chinese General Population: A Cross‐Sectional Study

**DOI:** 10.1111/1753-0407.70061

**Published:** 2025-03-02

**Authors:** Qingzheng Wu, Yu Cheng, Hongyan Liu, Yuepeng Wang, Bing Li, Yiming Mu

**Affiliations:** ^1^ Department of Endocrinology The First Medical Center of Chinese PLA General Hospital Beijing China; ^2^ School of Medicine Nankai University Tianjin China

**Keywords:** advanced glycation end products, coronary heart disease, REACTION study, skin autofluorescence

## Abstract

**Objective:**

The aim of this study was to investigate the relationship between SAF and CHD in the general population of China and to assess the feasibility of SAF used as a predictor of CHD.

**Methods:**

This study was nested within the prospective study REACTION (Cancer Risk Assessment in Chinese Diabetic Population) which included a total of 5806 eligible participants from two communities located in urban Beijing in 2018. SAF were measured using a fluorescence detector (DM Scan). CHD was the study endpoint and was determined by a face‐to‐face clinical survey. Pearson's correlation analysis, linear regression analysis, and binary logistic regression analysis were used to examine the association between SAF and CHD.

**Results:**

The overall prevalence of CHD in the general population was 12.1%. Logistic analysis showed that after full adjustment for confounding factors, the risk of CHD increased significantly with increasing lnSAF quartiles (*p*‐trend < 0.05). Compared to Q1 group, the multivariate adjusted ORs of Q2 and Q3 groups were 1.071 (0.817, 1.404), 1.025 (0.781, 1.344), respectively, and the OR was markedly increased at Q4 (OR = 1.377 [1.043, 1.817]). When lnSAF was a continuous variable, the risk of CHD increased with the elevation of lnSAF level. Stratified analysis showed that in subgroups with overweight (24–28 kg/m^2^), eGFR < 60 mL/min/1.73 m^2^, and diabetes mellitus (DM), lnSAF was still significantly correlated with CHD.

**Conclusions:**

In Chinese general population, higher lnSAF is independently associated with increased risk of CHD, and noninvasive SAF holds the potential to be a biomarker for CHD risk evaluation and stratification.

**Summary**

- SAF, a fluorescent substance of skin AGEs, can be used as a marker for noninvasive detection of human AGEs levels.
- In this cross‐sectional study, we found a significantly independent association between lnSAF and CHD, and the risk of CHD increased with the elevation of lnSAF in Chinese general population.
- To the best of our knowledge, this is the first large‐sample epidemiological study on the relationship between SAF and CHD in the general population of China.

## 1 Introduction

Coronary heart disease (CHD) is the major cause of death and the largest single contributor to mortality worldwide, generating huge global economic burden [1, 2]. The prevalence of CHD is on the rise and it is estimated that there are currently 11.39 million people suffering from CHD in China [3]. Atherosclerosis (AS) is the key pathological basis of CHD. As is a process that lipids, inflammatory cells, calcification, and other substances accumulate within the vascular wall, leading to the thickening and hardening of the vascular wall, and ultimately the formation of plaques [4, 5].

Advanced glycation end products (AGEs) is a class of complexes formed by the nonenzymatic reaction of glucose or other reducing sugars with biomolecules such as proteins, nucleic acids, or lipids [6, 7]. Previous studies has found that AGEs are closely related to physiological aging, age‐related diseases, diabetes, cardiovascular diseases (CVDs), and kidney diseases [8, 9]. AGEs seem to play an important role for the development and/or progression of CHD mainly through induction of oxidative stress and inflammation [10] and more effective management of circulating AGE levels could potentially contributes to slowing the progression of AS in patients with CHD [11].

AGEs such as pentosidine and methylglyoxal lysine dimer in skin tissues have autofluorescent properties [12], and the level of fluorescent AGEs in skin tissues can be detected using fluorometry [13, 14]. Studies have shown that in vitro skin autofluorescence (SAF) detection is highly consistent with the level of AGEs detected by skin tissue biopsy and with serum AGEs at that location [14, 15]. SAF detection does not require fasting, and possess some unique advantages such as low operating cost, noninvasive, rapid and safe detection, making it more suitable for large‐scale epidemiological investigation. In a prospective cohort study, de Vos et al. found that patients with peripheral vascular diseases had higher SAF values [16]. Multiple studies have showed that increased SAF can be considered as an indicator of extensive AS [17, 18]. However, there are some inconsistencies in existing research about SAF detection values and their disease relevance among different skin‐colored populations, and most existing studies focus on Caucasians and in specific groups such as DM or chronic kidney diseases (CKDs), while large‐sample studies on general populations in Asia are limited. Therefore, the aim of this study is to investigate the association between SAF and CHD and examine the feasibility of SAF as a predictor of CHD risk in the general population of China.

## 2 Materials and Methods

### 2.1 Study Population and Design

The present study was nested in the REACTION (Risk Evaluation of Cancers in Chinese Diabetic Individuals) study, which was designed to assess the correlation of diabetes and prediabetes with the risk of cancer in the Chinese population based on the community. The study population comprised permanent residents from three urban communities in Beijing center. The study protocol was approved by the Committee on Human Research at the Chinese People's Liberation Army (PLA) General Hospital, and written informed consent was obtained from all participants before data collection. For our study, 6527 participants were recruited in central Beijing between September and December 2018.

#### 2.1.1 Exclusion Criteria

Individuals diagnosed with end‐stage renal disease, mental illness, severe liver dysfunction, or advanced malignant tumor; participants aged ≤ 18 years old, lacking important data such as accurate blood glucose related indexes, SAF, BMI, and a documented history of drinking, smoking, hyperlipidemia, hypertension, T2DM, and so forth. Finally, a total of 5806 participants were included.

### 2.2 Measurement of SAF

An AGEs fluorescence detector (DM Scan, Hefei Institutes of Physical Science, Chinese Academy of Sciences) was applied by trained staff to detect the fluorescence values of skin AGEs, with a test range of 0 –150 AU, intra‐ and inter‐batch CVs < 3.0%. This is an assay based on SAF spectroscopy, which measures AGEs in the skin by fluorescence properties (excitation at 300–420 nm and emission at 420–600 nm). A 1–4 cm portion of the forearm was irradiated using an excitation light source with a peak wavelength of 370 nm. The subject's left arm was placed on the SAF fluorometer. Topical creams or lotions were discontinued 12 h prior to the examination, the test site avoided scarred, mossy sclerotic, and deformed skin, and the participant was instructed to remain motionless for the duration of the acquisition time (approximately 30 s). SAF was calculated based on the ratio between emitted and reflected light as measured by DM Scan. The mean value was obtained after three automatic measurements. The SAF values were natural logarithmically transformed, and the transformed lnSAF quartiles were as follows: Quartile 1 (Q1): 0–4.237; Quartile 2 (Q2): 4.237–4.320; Quartile 3 (Q3): 4.320–4.413; Quartile 4 (Q4): ≥ 4.413.

### 2.3 Data Collection

Data were collected by trained staff and nurses according to standardized procedures, including detailed questionnaires, routine physical examination. The questionnaires included age, gender, smoking status, drinking status, medical history, family medical history, and medication history. Subjects who smoked at least one cigarette per day or at least seven cigarettes per week in the past 6 months were defined as current smokers, and those who regularly drank alcohol once a week in the past 6 months were defined as current drinkers. Family medical history included first‐degree relatives. Height, weight, waist circumference (WC), and hip circumference (HC) were measured in a standard standing position, wearing light clothing without shoes, and recorded to one decimal place. Body mass index (BMI) was calculated as weight divided by the square of height (kg/m^2^). Waist‐to‐hip ratio (WHR) was calculated as WC divided by HC. Blood pressure was measured in a standard sitting position, three times every 5 min, and the mean values were taken for statistical analysis.

### 2.4 Laboratory Measurements

Blood samples were collected early in the morning after fasting at least 10 h. The biochemical markers such as fasting blood glucose (FBG), postprandial blood glucose (PBG), total cholesterol (TC), serum triglycerides (TG), high‐density lipoprotein cholesterol (HDL‐C), low‐density lipoprotein cholesterol (LDL‐C), alanine aminotransferase (ALT), aspartate aminotransferase (AST), fasting insulin, glycated hemoglobin (HbA1c), and serum creatinine (CREA) were measured using the fully automatic analyzer (Cobas 8000 Modular Analyzer Series; Roche Diagnostics, Basel, Switzerland) in the laboratory of Chinese PLA General Hospital followed by the laboratory standards. Non‐diabetic patients underwent a 75 g oral glucose tolerance test, with venous blood drawn at 0 and 120 min. Fasting plasma insulin levels were determined by the glucose oxidase‐peroxidase method. Estimated glomerular filtration rate (eGFR) was estimated using the CKD‐EPI formula updated by Inker et al. [19] in 2021.

### 2.5 Definition of Variables

Overweight was defined as 24 kg/m^2^ ≤BMI < 28 kg/m^2^, obesity was defined as BMI ≥ 28 kg/m^2^. Hypertension was defined as self‐reported hypertension and/or an average systolic blood pressure (SBP) ≥ 140 mmHg, and/or diastolic blood pressure (DBP) ≥ 90 mmHg over three measurements. Prehypertension was defined as an average SBP of 130–139 mmHg and/or DBP of 80–89 mmHg over three measurements. Diabetes was defined as self‐reported diabetes and/or newly diagnosed diabetes (FBG ≥ 7.0 mmol/L and/or PBG ≥ 11.1 mmol/L and/or HbA1c ≥ 6.5%). Pre‐diabetes is divided into the following three subgroups: Impaired Fasting Glucose (IFG): 6.1 ≤ FBG < 7.0 mmol/L and PBG < 7.8 mmol/L; Impaired Glucose Tolerance (IGT): FBG < 6.1 mmol/L and 7.8 ≤ PBG < 11.1 mmol/L; IFG + IGT: 6.1 ≤ FBG < 7.0 mmol/L and 7.8 ≤ PBG < 11.1 mmol/L; excluding self‐reported diabetes. Normal glucose tolerance population: No history of diabetes and FBG < 6.1 mmol/L and PBG < 7.8 mmol/L. Dyslipidemia was defined as self‐reported hyperlipidemia and/or TC ≥ 6.2 mmol/L and/or TG ≥ 2.3 mmol/L and/or LDL‐C ≥ 4.1 mmol/L and/or HDL‐C < 1.0 mmol/L.

### 2.6 Definition of CHD

CHD events were defined as self‐reported history of CHD by the subjects and/or definitive diagnosis of CHD based on clinical symptoms, Electrocardiograms, Coronary Computed Tomography Angiography, Coronary Angiography, Treadmill Exercise Test and other diagnostic criteria. Clinical outcomes were defined according to the International Classification of Diseases, eleventh Revision (ICD‐11) [20].

### 2.7 Statistical Methods

All statistical analyses were performed using SPSS 27.0 (IBM, Chicago, IL, USA). The data were divided into continuous and categorical variables, expressed as mean and standard deviation (SD) for normal distribution, median and interquartile range (IQR) for skewness, and frequency or percentage for categorical variables. The ANOVA test was used for continuous variables and the chi‐squared test was used for categorical variables for comparison between groups in this analysis. The SAF values were transformed by natural logarithm, and the lnSAF was in accordance with the normal distribution. We used multiple linear regression and Pearson's correlation analysis to investigate cross‐sectional associations between covariates and lnSAF, respectively. Logistic regression was used to analyze the associations between lnSAF and CHD by calculating the ratio of ratios (OR) and their 95% confidence intervals (CI). Three models with increasing adjustments were applied as follows: Model 1, adjusted for age, sex, BMI, current drinking, current smoking; Model 2, additionally adjusted for TC, TG, LDL‐C, SBP, DBP, HbA1c, eGFR; Model 3, additionally adjusted for DM (yes/no), hypertension (yes/no), dyslipidemia (yes/no), antidiabetic agents (yes/no), antihypertension agents (yes/no), lipid‐lowering agents (yes/no). We conducted subgroup analyses of the final model based on age (< 60 years, ≥ 60 years), gender (male, female), BMI (< 24, 24–28, ≥ 28 kg/m^2^), eGFR (< 60, 60–90, ≥ 90 mL/min/1.73 m^2^), glycemic status (normal blood glucose, pre‐diabetes, diabetes), blood pressure status (normal blood pressure, pre‐hypertension, hypertension), and dyslipidemia status (yes, no). Prediabetes was further stratified into IFG, IGT, and IFG + IGT. Potential interactions between LnSAF and stratification factors were evaluated.

All statistical tests were two‐sided, and *p* < 0.05 was considered statistically significant.

## 3 Result

### 3.1 Basic Clinical Characteristics of the Study Population

A total of 5806 participants were included in the analysis. All study subjects were divided into four groups based on the quartile of measured lnSAF. Table 1 shows the baseline characteristics of the study population across lnSAF quartiles. Participants were mostly female and median age was 62 years old. Compared with those in the lowest quartile of lnSAF, participants in the highest quartile of lnSAF were older, more likely to be current smokers or current drinkers, and had higher levels of SBP, FBG, PBG, and HbA1c, and had greater proportion of patients with CHD, DM, hypertension. Meanwhile, participants in the highest quartile of lnSAF had lower levels of eGFR, DBP, TC, TG, and LDL‐C, and smaller proportion of dyslipidemia than those in the lowest quartile (all *p* < 0.001).

**TABLE 1:** Basic characteristics of participants by lnSAF quartile.

	Total (*n* = 5806)	Q1 (*n* = 1472)	Q2 (*n* = 1445)	Q3 (*n* = 1448)	Q4 (*n* = 1441)	*p*
Age, years	62 (58, 66)	59 (55, 63)	61 (57, 65)	63 (59, 67)	65 (61, 72)	< 0.001
Male, *n* (%)	1969 (33.9%)	377 (25.6%)	395 (27.3%)	494 (34.1%)	703 (48.8%)	< 0.001
BMI, kg/m^2^	25.0 (23.0, 27.2)	25.4 (23.3, 27.6)	25.1 (23.1, 27.4)	24.8 (22.7, 27.1)	24.9 (22.8, 27.0)	< 0.001
WHR	0.89 (0.86, 0.93)	0.89 (0.85, 0.93)	0.89 (0.85, 0.93)	0.90 (0.86, 0.93)	0.90 (0.86, 0.94)	< 0.001
SBP, mmHg	132 (122, 144)	131 (120, 140)	131 (121, 143)	133 (122, 144)	135 (124, 147)	< 0.001
DBP, mmHg	79 (72, 85)	80 (73, 86)	79 (73, 85)	78 (72, 85)	78 (71, 85)	< 0.001
FBG, mmol/L	5.6 (5.2, 6.4)	5.5 (5.1, 6.0)	5.5 (5.1, 6.2)	5.6 (5.2, 6.5)	5.8 (5.3, 7.2)	< 0.001
PBG, mmol/L	5.9 (3.7, 8.6)	4.98 (3.43, 7.47)	5.79 (3.67, 8.03)	6.12 (3.80, 8.91)	6.84 (4.15, 11.08)	< 0.001
HbA1c (%)	5.8 (5.5, 6.2)	5.7 (5.5, 6.1)	5.7 (5.5, 6.1)	5.7 (5.5, 6.3)	5.9 (5.5, 6.6)	< 0.001
TC, mmol/L	5.31 (4.64, 5.99)	5.39 (4.78, 6.05)	5.35 (4.70, 6.02)	5.27 (4.61, 5.95)	5.20 (4.48, 5.92)	< 0.001
TG, mmol/L	1.37 (0.99, 1.94)	1.45 (1.04, 2.02)	1.39 (0.99, 1.98)	1.36 (0.99, 1.93)	1.31 (0.92, 1.84)	< 0.001
HDL‐C, mmol/L	1.45 (1.25, 1.69)	1.43 (1.24, 1.67)	1.46 (1.26, 1.71)	1.45 (1.24, 1.70)	1.45 (1.24, 1.68)	0.180
LDL‐C, mmol/L	4.23 (3.19, 6.68)	4.89 (3.48, 7.33)	4.30 (3.23, 6.69)	4.06 (3.09, 6.41)	3.96 (2.97, 5.99)	< 0.001
CREA, μmol/L	61.0 (54.0, 71.0)	57 (52, 66)	59 (53, 68)	62 (54, 72)	66 (57, 77)	< 0.001
eGFR, mL/min/1.73 m^2^	99.52 (92.72, 103.91)	102.44 (97.85, 106.51)	100.31 (94.79, 104.32)	98.66 (91.95, 103.07)	95.72 (87.54, 100.58)	< 0.001
Current smoking, *n* (%)	814 (14%)	127 (8.6%)	151 (10.4%)	210 (14.5%)	326 (22.6%)	< 0.001
Current drinking, *n* (%)	605 (10.4%)	112 (7.6%)	125 (8.7%)	149 (10.3%)	219 (15.2%)	< 0.001
CHD, *n* (%)	703 (12.1%)	121 (8.2%)	148 (10.2%)	172 (11.9%)	262 (18.2%)	< 0.001
DM, *n* (%)	1679 (28.9%)	293 (19.9%)	349 (24.2%)	449 (31.0%)	588 (40.8%)	< 0.001
Hypertension, *n* (%)	3173 (54.7%)	721 (49%)	768 (53.1%)	790 (54.6%)	894 (62.0%)	< 0.001
Dyslipidemia, *n* (%)	4225 (72.8%)	1155 (78.5%)	1062 (73.5%)	1033 (71.3%)	975 (67.7%)	< 0.001
Insulin, *n* (%)	271 (4.7%)	26 (1.8%)	39 (2.7%)	82 (5.7%)	124 (8.6%)	< 0.001
Antidiabetic agents, *n* (%)	870 (15.0%)	116 (7.9%)	164 (11.3%)	244 (16.9%)	346 (24.0%)	< 0.001
Antihypertension agents, *n* (%)	2074 (35.7%)	466 (37.1%)	509 (35.2%)	522 (36.0%)	577 (40.0%)	< 0.001
Lipid‐lowering agents, *n* (%)	1162 (20.0%)	266 (18.1%)	286 (19.8%)	320 (22.1%)	290 (20.1%)	< 0.001

*Note:* Data are medians (interquartile ranges) for continuous variables or percentages for categorical variables.

Abbreviations: BMI, body mass index; CHD, coronary heart disease; CREA, creatinine; DBP, diastolic blood pressure; DM, diabetes mellitus; eGFR, estimated glomerular filtration rate; FBG, fasting blood glucose; HDL‐C, high‐density lipoprotein cholesterol; LDL‐C, low‐density lipoprotein cholesterol; PBG, postprandial blood glucose; SAF, skin autofluorescence; SBP, systolic blood pressure; TC, total cholesterol; TG, triglycerides; WHR, waist‐to‐hip ratio.

Table S1 shows the baseline characteristics of participants with or without CHD. The overall prevalence of CHD was 12.1% (703 cases). The median SAF of patients with and without CHD were 74.8 and 78.6 AU. The participants with CHD had significantly higher level of SAF that those without CHD. Compared with the non‐CHD group, the CHD group was mostly female, older, more likely to be smokers and drinkers, and had higher levels of BMI, SBP, FBG, PBG, HbA1c, and lower levels of TC, HDL‐C, LDL‐C and eGFR (all *p* < 0.001). Moreover, CHD population had higher proportion of DM, hypertension, and dyslipidemia than those without CHD.

### 3.2 Determinants of lnSAF

We used multiple linear regression analyses to examine the association between lnSAF CHD and risk factors (Table 2). The regression equation was significant, *F* = 227.165, *p* < 0.05. Multivariate linear regression showed that age, sex, and current smoking were positively correlated with lnSAF, while BMI and eGFR were negatively correlated with lnSAF (all *p* < 0.05). However, the leves of WHR and current smoking were not significantly associated with lnSAF. Taken together, these variables explained 21.4% of the variance in lnSAF.

**TABLE 2:** Determinants of lnSAF in multivariate regression analysis.

Factors	B	*β*	*t*	*p*	F	Adjusted *R* ^2^
Age	0.006	0.320	21.518	0.000	227.165	0.214
Sex	0.016	0.056	3.837	0.000		
WHR	0.016	0.007	1.168	0.576		
BMI	−0.005	−0.118	−9.510	0.000		
eGFR	−0.002	−0.130	−8.884	0.000		
Current drinking	0.007	0.015	1.146	0.252		
Current smoking	0.061	0.157	11.184	0.000		

Abbreviations: BMI, body mass index; eGFR, estimated glomerular filtration rate; WC, waist circumference; WHR, waist‐to‐hip ratio.

### 3.3 Association Between lnSAF and CHD

As shown in Table 3, after full adjustment for confounding factors, the risk of CHD increased significantly with increasing lnSAF quartiles (*p*‐trend < 0.05). Compared to Q1 group, the multivariate adjusted ORs of Q2 and Q3 groups were 1.071 (0.817, 1.404), 1.025 (0.781, 1.344), respectively, and the OR was markedly increased at Q4 (OR = 1.377 [1.043, 1.817]). When lnSAF was a continuous variable, the risk of CHD also increased with the elevation of lnSAF level.

**TABLE 3:** Association between lnSAF quartile and CHD in Chinese community adults.

	Model 1		Model 2		Model 3	
	OR (95% CI)	*p*‐trend	OR (95% CI)	*p*‐trend	OR (95% CI)	*p*‐trend
lnAGEs quartile		< 0.001		0.037		0.030
Q1	1.00		1.00		1.00	
Q2	1.137 (0.879, 1.470)		1.062 (0.817, 1.382)		1.071 (0.817, 1.404)	
Q3	1.208 (0.937, 1.558)		1.059 (0.815, 1.376)		1.025 (0.781, 1.344)	
Q4	1.618 (1.255, 2.087)^a^		1.325 (1.014, 1.732)^a^		1.377 (1.043, 1.817)^a^	
lnAGEs Continuous	3.216 (1.688, 6.125)	< 0.001	2.001 (1.007, 3.973)	0.048	2.145 (1.048, 4.389)	0.037

*Note:* Model 1: Adjusted for age, sex, BMI, current drinking, current smoking.

Model 2: Additionally adjusted for TC, TG, LDL‐C, SBP, DBP, HbA1c, eGFR.

Model 3: Additionally adjusted for DM (yes/no), hypertension (yes/no), dyslipidemia (yes/no), antidiabetic agents (yes/no), antihypertension agents (yes/no), lipid‐lowering agents (yes/no).

^a^ Significant data.

### 3.4 Association Between lnSAF and CHD in Stratified Analysis

As shown in Table 4, we conducted stratified analysis to verify the association between lnSAF and CHD. The results denoted that in subgroups with overweight (24–28 kg/m^2^), eGFR < 60 mL/min/1.73 m^2^, and DM, lnSAF was significantly correlated with CHD in Q4. In addition, in people with IGT, the risk of CHD increases significantly with the elevation of lnSAF quartiles (Table 5). Furthermore, no significant interaction was found between lnSAF and confounding factors.

**TABLE 4:** Association between lnSAF‐AGEs and CHD in stratified analysis.

	lnAGEs quartile				*p*‐trend	*p* for interaction
	Q1	Q2	Q3	Q4		
Total						
OR (95% CI)	1.00	1.274 (0.990, 1.639)	1.505 (1.178, 1.923)^a^	2.481 (1.973, 3.120)^a^	< 0.001	
Multivariable‐adjusted OR (95% CI)	1.00	1.071 (0.817, 1.404)	1.025 (0.781, 1.344)	1.377 (1.043, 1.817)^a^	0.019	
Age, years						0.325
< 60						
OR (95% CI)	1.00	1.012 (0.633, 1.616)	1.163 (0.709, 1.906)	2.449 (1.487, 4.033)^a^	0.002	
Multivariable‐adjusted OR (95% CI)	1.00	0.868 (0.519, 1.451)	0.854 (0.485, 1.504)	1.973 (1.079, 3.606)^a^	0.091	
≥ 60						
OR (95% CI)	1.00	1.188 (0.873, 1.616)	1.275 (0.949, 1.713)	1.835 (1.391, 2.421)^a^	< 0.001	
Multivariable‐adjusted OR (95% CI)	1.00	1.128 (0.812, 1.566)	1.093 (0.795, 1.505)	1.443 (1.052, 1.979)^a^	0.014	
Sex						0.893
Male						
OR (95% CI)	1.00	1.209 (0.7784, 1.866)	1.317 (0.875, 1.981)	2.055 (1.420, 2.973)^a^	< 0.001	
Multivariable‐adjusted OR (95% CI)	1.00	0.991 (0.619, 1.586)	1.004 (0.635, 1.588)	1.426 (0.909, 2.238)	0.052	
Female						
OR (95% CI)	1.00	1.294 (0.949, 1.765)	1.540 (1.132, 2.094)^a^	2.448 (1.810, 3.310)^a^	< 0.001	
Multivariable‐adjusted OR (95% CI)	1.00	1.122 (0.806, 1.564)	1.053 (0.751, 1.477)	1.378 (0.965, 1.969)	0.100	
BMI, kg/m^2^						0.133
< 24						
OR (95% CI)	1.00	0.882 (0.516, 1.508)	1.555 (0.975, 2.480)	2.107 (1.343, 3.306)^a^	< 0.001	
Multivariable‐adjusted OR (95% CI)	1.00	0.629 (0.353, 1.119)	0.924 (0.553, 1.545)	0.856 (0.497, 1.476)	0.983	
24–28						
OR (95% CI)	1.00	1.204 (0.834, 1.738)	1.440 (1.004, 2.066)^a^	2.811 (2.028, 3.896)^a^	< 0.001	
Multivariable‐adjusted OR (95% CI)	1.00	1.075 (0.725, 1.595)	1.023 (0.684, 1.529)	1.710 (1.152, 2.538)^a^	0.003	
≥ 28						
OR (95% CI)	1.00	1.940 (1.215, 3.098)	1.766 (1.089, 2.864)	2.668 (1.660, 4.287)	< 0.001	
Multivariable‐adjusted OR (95% CI)	1.00	1.567 (0.950, 2.584)	1.148 (0.674, 1.957)	1.391 (0.783, 2.473)	0.483	
eGFR, mL/min/1.73 m^2^						0.735
< 60						
OR (95% CI)	1.00	1.224 (0.922, 1.625)	1.511 (1.145, 1.994)^a^	2.293 (1.755, 2.997)^a^	0.147	
Multivariable‐adjusted OR (95% CI)	1.00	1.046 (0.773, 1.417)	1.071 (0.789, 1.455)	1.405 (1.023, 1.929)^a^	0.488	
60–90						
OR (95% CI)	1.00	1.104 (0.618, 1.972)	0.999 (0.575, 1.736)	1.559 (0.935, 2.597)	0.028	
Multivariable‐adjusted OR (95% CI)	1.00	0.906 (0.470, 1.748)	0.787 (0.421, 1.473)	1.125 (0.607, 2.085)	0.446	
≥ 90						
OR (95% CI)	1.00	1.145 (0.645, 2.034)	1.003 (0.580, 1.735)	1.680 (1.015, 2.779)^a^	0.008	
Multivariable‐adjusted OR (95% CI)	1.00	0.953 (0.501, 1.813)	0.801 (0.433, 1.470)	1.195 (0.657, 2.175)	0.309	
Glycemic status						0.298
Normal blood glucose						
OR (95% CI)	1.00	1.088 (0.755, 1.567)	1.254 (0.871, 1.805)	1.583 (1.096, 2.288)^a^	0.011	
Multivariable‐adjusted OR (95% CI)	1.00	0.838 (0.561, 1.251)	0.982 (0.651, 1.482)	0.926 (0.591, 1.449)	0.906	
Prediabetes						
OR (95% CI)	1.00	1.106 (0.564, 1.831)	1.490 (0.858, 2.587)	1.905 (1.123, 3.233)^a^	0.006	
Multivariable‐adjusted OR (95% CI)	1.00	1.011 (0.536, 1.907)	1.248 (0.672, 2.316)	1.641 (0.864, 3.118)	0.091	
Diabetes						
OR (95% CI)	1.00	1.646 (1.049, 2.583)^a^	1.552 (1.005, 2.396)^a^	2.799 (1.874, 4.181)^a^	< 0.001	
Multivariable‐adjusted OR (95% CI)	1.00	1.529 (0.944, 2.475)	1.140 (0.710, 1.830)	2.062 (1.288, 3.302)^a^	0.003	
Blood pressure status						0.659
Normal blood pressure						
OR (95% CI)	1.00	1.149 (0.536, 2.464)	1.350 (0.628, 2.899)	3.821 (1.934, 7.549)^a^	< 0.001	
Multivariable‐adjusted OR (95% CI)	1.00	1.009 (0.434, 2.343)	0.949 (0.404, 2.230)	1.642 (0.698, 3.862)	0.239	
Prehypertension						
OR (95% CI)	1.00	1.203 (0.694, 2.087)	1.237 (0.720, 2.125)	2.210 (1.335, 3.658)^a^	0.002	
Multivariable‐adjusted OR (95% CI)	1.00	1.040 (0.580, 1.867)	0.926 (0.514, 1.668)	1.348 (0.744, 2.443)	0.349	
Hypertension						
OR (95% CI)	1.00	1.265 (0.929, 1.723)	1.528 (1.133, 2.060)^a^	2.132 (1.610, 2.825)^a^	< 0.001	
Multivariable‐adjusted OR (95% CI)	1.00	1.067 (0.767, 1.485)	1.073 (0.772, 1.493)	1.332 (0.949, 1.870)	0.077	
Dyslipidaemia status						0.457
No						
OR (95% CI)	1.00	1.447 (0.735, 2.846)	2.245 (1.197, 4.211)^a^	3.383 (1.861, 6.152)^a^	< 0.001	
Multivariable‐adjusted OR (95% CI)	1.00	1.112 (0.546, 2.263)	1.361 (0.693, 2.675)	1.501 (0.762, 2.956)	0.179	
Yes						
OR (95% CI)	1.00	1.295 (0.985, 1.702)	1.447 (1.105, 1.895)^a^	2.512 (1.951, 3.234)^a^	< 0.001	
Multivariable‐adjusted OR (95% CI)	1.00	1.060 (0.789, 1.424)	0.932 (0.690, 1.259)	1.353 (0.993, 1.842)	0.065	

*Note:* Adjusted for age, sex, BMI, TC, TG, LDL‐C, SBP, DBP, HbA1c, eGFR, current drinking, current smoking, DM (yes/no), hypertension (yes/no), dyslipidemia (yes/no), antidiabetic agents (yes/no), antihypertension agents (yes/no), lipid‐lowering agents (yes/no).

Abbreviations: BMI, body mass index; eGFR, estimated glomerular filtration rate; OR, odds ratio; SAF, skin autofluorescence.

^a^ Significant data.

**TABLE 5:** Association between lnSAF‐AGEs and CHD in prediabetes subgroup.

Prediabetes	lnAGEs quartile				*p* trend	*p* for Interaction
	Q1	Q2	Q3	Q4		
IFG						
OR (95% CI)	1.00	0.984 (0.395, 2.453)	0.991 (0.375, 2.618)	1.026 (0.373, 2.824)	0.963	0.536
Multivariable‐adjusted OR (95% CI)	1.00	1.142 (0.288, 4.537)	1.127 (0.336, 3.777)	1.210 (0.391, 3.745)	0.860	
IGT						
OR (95% CI)	1.00	1.309 (0.565, 3.031)	1.575 (0.696, 3.565)	2.674 (1.273, 5.619)^a^	0.005	
Multivariable‐adjusted OR (95% CI)	1.00	1.118 (0.464, 2.695)	1.237 (0.513, 2.983)	2.250 (0.938, 5.396)	0.046	
IFG + IGT						
OR (95% CI)	1.00	0.267 (0.026, 2.730)	2.222 (0.554, 8.920)	1.730 (0.417, 7.177)	0.177	
Multivariable‐adjusted OR (95% CI)	1.00	0.221 (0.007, 6.623)	1.320 (0.124, 14.082)	1.736 (0.143, 21.078)	0.407	

*Note:* Adjusted for age, sex, BMI, TC, TG, LDL‐C, SBP, DBP, HbA1c, eGFR, current drinking, current smoking, DM (yes/no), hypertension (yes/no), dyslipidemia (yes/no), antidiabetic agents (yes/no), antihypertension agents (yes/no), lipid‐lowering agents (yes/no).

Abbreviations: BMI, body mass index; eGFR, estimated glomerular filtration rate; OR, odds ratio; SAF, skin autofluorescence.

^a^ Significant data.

## 4 Discussion

In this cross‐sectional study, we found a significantly independent association between lnSAF and CHD and the risk of CHD increased with the elevation of lnSAF in Chinese general population. Further stratified analysis showed that the association was significant in those with overweight (24–28 kg/m^2^), eGFR < 60 mL/min/1.73 m^2^, and DM. To the best of our knowledge, this is the first large‐sample epidemiological study on the relationship between SAF and CHD in the general population of China.

In recent years, some studies have begun to focus on the relationship between AGEs and CVD in the general population, but those studies yielded inconsistent results. For example, Hanssen et al. [21] in two Dutch cohort studies (*n* = 1291) found no independent adverse associations between plasma AGEs and CVD risk in individuals with and without diabetes, while Lamprea et al. [22] found that α‐dicarbonyl‐derived AGEs were associated with CVD in American old population. In African area, Kerkeni et al. [23] found in their study of 101 patients undergoing coronary angiography that circulating pentosidine (a type of AGE) levels were independently associated with the development of CHD and correlated with the severity of CHD, irrespective of the diabetic status. Kilhovd et al. [24] used immunoassay to measure serum AGEs in a random sample of 1141 middle‐aged, non‐diabetic Finns and found that serum AGEs measured at baseline predicted CHD mortality in women 18 years later. Studies on the relationship between AGE accumulation as reflected by SAF and CHD have also yielded valuable results. The LifeLines Cohort Study [25] including 3839 participants showed that SAF is associated with the degree of coronary calcification in the general population. Kawamoto et al. [26] reported in a single‐center prospective study that, SAF was independently associated with cardiovascular events (CVEs) among patients with coronary artery disease treated with PCI. In addition, a previous study by our group identified the association between SAF AGEs and the prevalence of ear lobe crease [27], indirectly suggesting a close relationship between SAF and CHD. In the present study, we found for the first time in Chinese general population that there was a significantly independent correlation between SAF and CHD. This finding implies that in the Chinese population, SAF could serve as a novel biomarker for CHD and SAF detection is conducive to early identification and intervention of individuals at high risk of CHD, which may help to reduce the prevalence of CHD. Nevertheless, additional large‐scale prospective studies are needed to verify this association.

The positive correlation between SAF and CHD may be associated with various biological mechanisms. First, AGEs could activate inflammatory signaling pathways such as NF‐κB through receptor RAGE and non‐receptor pathways, which lead to increased expression and release of inflammatory cytokines (such as IL‐6 and TNF‐α) that play a key role in the inflammatory process of CHD [9, 28, 29, 30]. Second, AGEs increase the risk of AS by promoting the proliferation and migration of vascular smooth muscle cells (VSMCs) and by inducing the deposition of extracellular matrix, leading to the thickening and hardening of vessel wall [31]. Third, the formation of AGEs is accompanied by the generation of reactive oxygen species (ROS) which can damage vascular endothelial cells and reduce the bioavailability of nitric oxide (NO), leading to impaired vasodilation [32, 33, 34]. Additionally, AGEs may also be related to the development of well‐known CHD risk factors including insulin resistance and metabolic syndrome [35]. Furthermore, experimental study in mouse models [36, 37] showed that lowering or blocking AGEs attenuated plaque formation in aortas and clinical studies in diabetic patients demonstrated that inhibiting or blocking AGEs attenuated AS [38]. Thus, AGEs and RAGE represent potential therapeutic targets for CHD in the future.

Previous studies have demonstrated the association of AGEs with CVD in diabetic populations [39, 40, 41, 42, 43, 44]. Consistently, we also found a significant correlation between SAF and CHD among individuals with DM. Surprisingly, we showed for the first time that in IGT subgroup, the risk of CHD increases with the rising SAF quartile, indicating that more attention should be paid to SAF levels in IGT population. However, the association between SAF and CHD in the IGT population was not significant, which needs further validation in larger‐scale population. In addition, we found a correlation between SAF and CHD in the overweight population, while no similar association was found in obese populations. This is a novel discovery of this research. It may be attributed to the fact that other prevalent risk factors (such as hypertension and dyslipidemia) in the obese population may overshadow the impact of SAF on CHD, and further researches are needed to explore the specific mechanisms behind these associations. Anyhow, our results suggested the importance of stratified management of CHD based on weight and it is necessary to detect SAF levels in overweight population to early identify and prevent CHD.

For the study of the relationship between AGEs and CHD, the population with CKD has received the most attention besides the diabetic population. Although it remains controversial whether SAF can predict the decline of renal function, studies on the association between SAF and the risk of CHD in CKD patients, especially in those on dialysis, have been widely conducted and have yielded valuable results. A meta‐analysis [45] including nine studies showed that the SAF levels were associated with higher risk of cardiovascular morbidity, cardiovascular and overall mortalities in hemodialysis (HD) patients. In addition, Shardlow et al. [46] identified SAF as an independent risk factor for CVE in patients with early stage CKD in a prospective study of patients with CKD stage 3 (eGFR = 30–59 mL/min/1.73 m^2^). Consistently, our study excluded patients with end‐stage renal disease and those on dialysis (eGFR < 30 mL/min/1.73 m^2^). We found that SAF is significantly associated with the risk of CHD at Q4 in the population with an eGFR < 60 mL/min/1.73 m^2^. The above studies collectively suggested that in populations with CKD, focusing on SAF levels may contribute to the prevention of CHD.

Our study has some limitations. First, the measurement of SAF is not comprehensive for AGEs analysis: not all AGEs are fluorescent in nature. However, SAF, as a noninvasive method, was confirmed to be strongly consistent with AGEs in tissue and serum in validation studies, and SAF remained stable over time compared with plasma AGEs. Nonetheless, the absence of data regarding former smoking, time since smoking cessation, and coffee consumption constitutes a significant limitation. Studies [47, 48] have reported that current smoking, pack‐years of smoking, and coffee consumption can significantly affect SAF levels, which may consequently influence our research outcomes. In future studies, attention should be paid to collecting relevant data to mitigate the impact of confounding factors on the results. In addition, most of the previous studies were conducted in western white people, and the skin color can affect the measurement of SAF. In this study, we applied the DM Scan [49], a self‐developed AGEs fluorescence detector for yellow skin developed by Hefei Institutes of Physical Science, Chinese Academy of Sciences, to make the measurement of SAF more accurate. Second, the respondents were from two communities located in downtown Beijing, China, and the results obtained from these individuals are at least generalizable to the broader Beijing and Chinese community general population. However, a wide range of multicenter studies are needed to confirm the generalizability of our findings. Third, this is a cross‐sectional study, which cannot allow causal inferences or rule out residual confounders, so our results need to be further validated in larger prospective studies.

## 5 Conclusion

In conclusion, we found that higher SAF was independently associated with an increased risk of CHD in the general Chinese population, indicating the potential of noninvasive SAF as a biomarker to incorporate into CHD risk stratification. In addition, SAF detection may serve as an effective and convenient tool to evaluate the risk of CHD and screen high‐risk populations in clinical work or large‐scale epidemiological surveys.

## Author Contributions

Qingzheng Wu and Yu Cheng contributed to the conception, design, data analyses and drafting of the manuscript, who were major contributors in writing the manuscript. Hongyan Liu contributed to the concept and drafting of the manuscript. Yuepeng Wang contributed to data collection, collation and analyses. Yiming Mu and Bing Li were the superior advisors and they were responsible for the design and supervision of the manuscript.

## Ethics Statement

The research program was authorized by the Human Research of Rui‐Jin Hospital affiliated with the School of Medicine of Shanghai Jiao Tong University. Written informed consents were obtained from all participants before data collection (No. 2011–14).

## Conflicts of Interest

The authors declare no conflicts of interest.

## Supporting information


**Data S1.** Supporting Information.

## Data Availability

Further inquiries can be addressed to the corresponding author.
