# Supplementary material for: Association Between Skin Autofluorescence and Coronary Heart Disease in Chinese General Population: A Cross‐Sectional Study
Source: J Diabetes. 2025 Mar 2;17(3):e70061. doi: 10.1111/1753-0407.70061 (PMC11872386; doi:10.1111/1753-0407.70061)
Supplement: Supplementary file 1 — Data S1. Supporting Information. [file JDB-17-e70061-s001.docx]

Supplementary Table 1 Distribution of risk factors in relation to CHD.

| Variable | Total  (n = 5806) | Non-CHD  (n = 5103) | CHD  (n = 703) | *P*-value |
| --- | --- | --- | --- | --- |
| Age, y | 62 (58, 66) | 61 (57, 66) | 65 (61, 71) | <0.001 |
| Sex, n (%) | 1969 (33.9%) | 1661 (32.5%) | 308 (43.8%) | <0.001 |
| BMI, kg/m^2^ | 25.0 (23.0, 27.2) | 25.0 (23.0, 27.1) | 25.9 (24.0, 28.0) | <0.001 |
| WHR | 0.89 (0.86, 0.93) | 0.89 (0.85, 0.93) | 0.90 (0.87, 0.95) | <0.001 |
| SBP, mm Hg | 132 (122, 144) | 132 (121, 143) | 134 (124, 145) | <0.001 |
| DBP, mm Hg | 79 (72, 85) | 79 (73, 85) | 77 (71, 84) | <0.001 |
| FBG, mmol/L | 5.6 (5.2, 6.4) | 5.5 (5.1, 6.2) | 6.0 (5.4, 7.6) | <0.001 |
| PBG, mmol/L | 5.9 (3.7, 8.6) | 5.83 (3.73, 8.38) | 6.90 (3.23, 11.36) | <0.001 |
| HbA1c (%) | 5.8 (5.5, 6.2） | 5.7 (5.4, 6.2) | 6.1 (5.7, 7.0） | <0.001 |
| TC, mmol/L | 5.31(4.64, 5.99) | 5.37 (4.75, 6.05) | 4.67 (4.04, 5.56) | <0.001 |
| TG, mmol/L | 1.37 (0.99, 1.94) | 1.37 (0.98, 1.95) | 1.38 (1.01, 1.88) | 0.754 |
| HDL-C, mmol/L | 1.45 (1.25, 1.69) | 1.46 (1.26, 1.70) | 1.37 (1.18, 1.58) | <0.001 |
| LDL-C, mmol/L | 4.23 (3.19, 6.68） | 4.28 (3.26, 6.64) | 3.74 (2.56, 7.20) | <0.001 |
| CREA, umol/L | 61.0 (54.0, 71.0) | 60 (53, 70) | 65 (56, 76) | <0.001 |
| eGFR, mL/min/1.73m^2^ | 99.52 (92.72, 103.91） | 99.94 (93.47, 104.17) | 96.43 (87.91, 101.23) | <0.001 |
| lnSAF | 4.330±0.136 | 4.324±0.135 | 4.371±0.137 | <0.001 |
| SAF, AU | 75.2 (69.2, 82.5) | 74.8 (68.9, 81.9) | 78.6 (71.9, 87.1) | <0.001 |
| Current smoking, n (%) | 814 (14%) | 702 (13.8%) | 112 (15.9%) | <0.001 |
| Current drinking, n (%) | 605 (10.4%) | 526 (10.3%) | 79 (11.2%) | <0.001 |
| DM, n (%) | 1679 (28.9%) | 1349 (26.4%) | 330 (46.9%) | <0.001 |
| Hypertension, n (%) | 3173 (54.7%) | 2668 (52.3%) | 505 (71.8%) | <0.001 |
| Dyslipidemia, n (%) | 4225 (72.8%) | 3662 (71.8%) | 563 (80.1%) | <0.001 |
| Antidiabetic agents, n (%) | 870 (15.0) | 655 (12.8%) | 215 (30.6%) | <0.001 |
| Insulin, n (%) | 271 (4.7%) | 169 (3.3%) | 102 (14.5%) | <0.001 |
| Antihypertension agents, n (%) | 2074 (35.7%) | 1650 (32.3%) | 424 (60.3%) | <0.001 |
| Lipid-lowering agents, n (%) | 1162 (20.0%) | 826 (16.2%) | 336 (47.8%) | <0.001 |

Data are medians (interquartile ranges) for continuous variables or percentages for categorical variables.

Abbreviations: BMI, body mass index; WHR, waist-to-hip ratio; SBP, systolic blood pressure; DBP, diastolic blood pressure; FBG, fasting blood glucose; PBG, postprandial blood glucose; TC, total cholesterol; TG, triglycerides; LDL-C, low-density lipoprotein cholesterol; HDL-C, high-density lipoprotein cholesterol; CREA, creatinine; eGFR, estimated glomerular filtration rate; CHD, coronary heart disease; DM, diabetes mellitus.
